# Generating GPS activity spaces that shed light upon the mobility habits of older adults: a descriptive analysis

**DOI:** 10.1186/1476-072X-13-51

**Published:** 2014-12-12

**Authors:** Jana A Hirsch, Meghan Winters, Philippa Clarke, Heather McKay

**Affiliations:** Centre for Hip Health and Mobility and Department of Medicine, University of British Columbia, 2635 Laurel Street, Vancouver, British Columbia V5Z 1 M9 Canada; Carolina Population Center, University of North Carolina at Chapel Hill, 206 West Franklin St, Chapel Hill, NC 27516 USA; Faculty of Health Sciences, Simon Fraser University, 8888 University Drive, Burnaby, British Columbia V5A 1S6 Canada; Institute for Social Research, University of Michigan, 426 Thompson Street, Ann Arbor, MI 48104 USA; Centre for Hip Health and Mobility and Department of Family Practice, University of British Columbia, 2635 Laurel Street, Vancouver, British Columbia V5Z 1 M9 Canada

**Keywords:** Global positioning systems (GPS), Geographic information systems (GIS), Activity space, Mobility, Neighborhood attributes, Older adults

## Abstract

**Background:**

Measuring mobility is critical for understanding neighborhood influences on older adults’ health and functioning. Global Positioning Systems (GPS) may represent an important opportunity to measure, describe, and compare mobility patterns in older adults.

**Methods:**

We generated three types of activity spaces (Standard Deviation Ellipse, Minimum Convex Polygon, Daily Path Area) using GPS data from 95 older adults in Vancouver, Canada. Calculated activity space areas and compactness were compared across sociodemographic and resource characteristics.

**Results:**

Area measures derived from the three different approaches to developing activity spaces were highly correlated. Participants who were younger, lived in less walkable neighborhoods, had a valid driver’s license, had access to a vehicle, or had physical support to go outside of their homes had larger activity spaces. Mobility space compactness measures also differed by sociodemographic and resource characteristics.

**Conclusions:**

This research extends the literature by demonstrating that GPS tracking can be used as a valuable tool to better understand the geographic mobility patterns of older adults. This study informs potential ways to maintain older adult independence by identifying factors that influence geographic mobility.

**Electronic supplementary material:**

The online version of this article (doi:10.1186/1476-072X-13-51) contains supplementary material, which is available to authorized users.

## Background

Mobility is defined as the “ability to move oneself (either independently or by using assistive devices or transportation) within environments that expand from one’s home to one’s neighborhood and regions beyond” [1]. Mobility is key to older adults leading active, healthy, independent lives [2] and is central to them conducting commercial, cultural, and social activities [3–5]. Older adults’ well-being and quality of life are also closely linked to their mobility [5–8]. The demographic shift toward an aging population is unprecedented in western society and demands novel solutions that evaluate and promote the mobility of older adults. These solutions may be embedded within transportation systems and planning [9–13].

It is essential to identify effective tools to describe older adult mobility so as to better understand the influence of neighborhood on their health and mobility [14]. “Life-space” is a frequently used measure of older adult mobility [15–17]. This self-reported measure of the extent of recent travel (using thresholds such as: within the home, into the local neighborhood, or beyond) was positively associated with diminished cognitive decline and Alzheimer’s Disease [18, 19], lower risk of both death [20] and becoming more frail [21]. Older age, being female, and having physical limitations have been associated with smaller life-spaces, as is having had a stroke, high depressive symptoms, and being obese [22, 23]. Higher education, better lower extremity function and muscle strength were associated larger life-spaces [22, 23]. Importantly, the ability to drive plays a key role in the mobility of older adults as captured using life-space measures [23–26].

Global Positioning Systems (GPS) technology may provide a means to calculate geographic range as a measure of mobility. GPS has been used to objectively characterize life-space [27–32] and also to detect outside physical activity [33]. Most research using GPS for older adult mobility comes from one study—the Senior Tracking (SenTra) project, based in Germany and Israel. SenTra used GPS points or metrics of out-of-home behaviors (e.g. number of visited places, time spent outside the home, and distance traveled from home) to assess mobility patterns in a sample of older adults who were cognitively impaired or had Alzheimer’s disease, and compared this clinical group with community-dwelling older adults [34–47]. It may not be possible to generalize outcomes from SenTra across geographically and culturally diverse settings, or with older adults who are independent and able to make their own travel decisions. Therefore, there is a need to investigate GPS applications in community-dwelling older adults in the North American context, given the limited knowledge base on measurement approaches to define the geographic patterns (i.e. shape) and extent (i.e. size) of older adult mobility in this population. Additionally, the potential to characterize older adults’ geographic extent or pattern was not explored in any of these studies, leaving questions about where older adults travel.

To represent daily mobility, neighborhood studies of physical activity have used GPS-based “activity spaces” as an individual-based measure of spatial behavior [48–52]. Activity spaces differ from the life-space measure, in that they focus on neighborhood (out of home) behavior only, rather than mobility both within and beyond the home. Additionally, with the exception of recent efforts to incorporate GPS [27–32], the majority of life-space studies are based on self-reported travel extents (within the home, into the local neighborhood, or beyond), not spatially-located travel data. Thus, the expansion of activity spaces to the investigation of older adult mobility will give additional insight into the community factors and resources that shape neighborhood activity. It is hypothesized that activity spaces may vary in size and shape across different populations, such as those with low incomes or different age groups [50, 53]. To date the utility of GPS to measure, describe, and compare mobility patterns in older adults has not been fully explored. Previous work proposed using a “mobility envelope”, the length of the outer perimeter of spatial excursions made by individuals, as an outcome measure for mobility studies in older adults [54]. By integrating various metrics of geographic extent from activity space studies, the mobility envelope concept could be advanced. For example, additional metrics evaluating different dimensions of individuals’ geographic scope may be useful for understanding mobility. In particular, in urban planning the shape or “compactness” of activity spaces is a metric of how circular a polygon is and is a concept thought to illustrate the capacity of neighborhoods to provide opportunities to “live, work, shop, and socialize at the local scale” [53]. Specifically, compactness has been shown to vary across different travel modes [55]. However, comparisons of compactness among different individuals (and within a particular type of activity space) may reveal important information on the role of driving in older adult mobility.

Therefore our objectives are twofold: to create and compare different types of geographic activity spaces for community dwelling older adults, so as to clarify the extent and pattern of their mobility; and then within each activity space approach, to assess individual sociodemographic and resource characteristics that are associated with larger (or smaller) or more (or less) compact activity spaces. We hypothesize that the size and shape of activity spaces will vary by the approach used to create them. Within each activity space type we hypothesize that participants who are younger, healthier, and with better access to transportation (driving or material resources for going outside) will have larger activity spaces. Furthermore, we hypothesize that those who drive less will have more compact activity spaces, and also that participants who live in more walkable neighborhoods will have more compact activity spaces, since amenities may be closer to home.

## Results

Participants had GPS data for a mean of 3.5 days (standard deviation (SD) 1.7 days; median 3.0 interquartile range (IQR) 3.0) with a mean of 14656.3 GPS points (SD 10232.8; median 12132, IQR 12401). Participants traveled between 1 and 12 trips each day they were tracked, with a mean of 13.2 total trips (SD 7.8; median 13.0, IQR 12.0) per participant.

**Figure 1:**
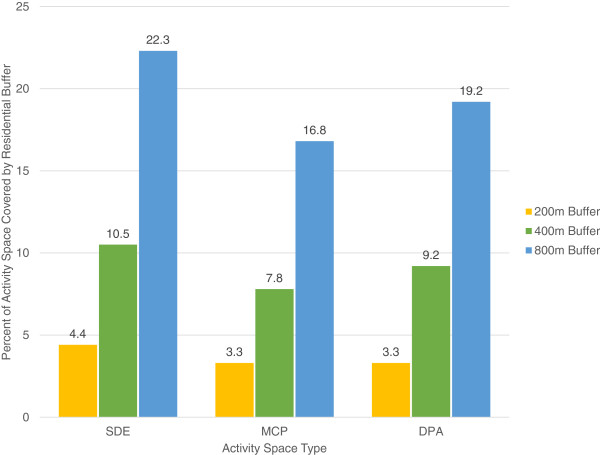
**Percentage of activity space covered by traditional residential buffers (200-meter, 400-meter, 800-meter).**

The areas derived from the three different approaches to developing activity spaces were highly associated; correlation coefficients ranged from ρ = 0.96 (Standard Deviation Ellipse (SDE) area vs Daily Path Area (DPA) area) to ρ = 0.98 (Minimum Convex Polygon (MCP) area vs DPA area and MCP area vs SDE area). However, values for compactness varied greatly between approaches; although compactness values derived from MCP and SDE approaches were highly correlated (ρ = 0.82, p < 0.0001), compactness values derived using DPA were not correlated with either MCP or SDE approaches (ρ = 0.07 p = 0.49 and ρ = −0.01 p = 0.92, respectively). Activity spaces were larger than the traditional buffers (200-meters or 1/8-mile; 400-meters or ¼-mile; 800-meters or ½-mile) used for neighborhood research (Figure 1).

In terms of size, DPA generated the smallest and MCP the largest (Table 1) activity space areas. Patterns for activity space area by sociodemographic group and resource characteristics were consistent across approaches. Activity space areas were generally larger for younger participants, those in less walkable neighborhoods, those with valid driver’s licenses, those with access to a vehicle in the past 7 days, and those with physical support to go outside.

**Table 1:** **GPS-based activity space areas of Walk The Talk Study participants (n = 95) by sociodemographic group and resource characteristics**

Sociodemographic group	n	SDE area (in hectares)		MCP area (in hectares)		DPA area (in hectares)	
		Median (IQR)	***p*** ^a^	Median (IQR)	***p*** ^a^	Median (IQR)	***p*** ^a^
All	95	1121.9 (3900.8)		1753.6 (7097.9)		837.2 (1389.1)	
Sex			0.3467		0.4616		0.6060
Female	63	1183.8 (4903.2)		1753.6 (7256.0)		837.2 (1603.6)	
Male	32	972.7 (2355.6)		1752.6 (4974.1)		839.8 (1147.7)	
Age (years)			0.0289		0.0617		0.1293
65-69	26	3071.1 (8827.8)		4764.5 (12583.2)		1448.6 (1588.1)	
70-74	29	1909.6 (3362.5)		1952.4 (6744.7)		958.4 (1150.5)	
75-79	26	680.5 (1423.0)		1031.3 (2325.3)		566.5 (937.2)	
80+	14	483.2 (1397.3)		791.8 (2624.7)		532.6 (548.3)	
Race			0.2727		0.3351		0.4617
Non-white	18	693.5 (5189.3)		1163.0 (5545.7)		786.4 (1486.7)	
White	77	1379.6 (3539.9)		1896.0 (6854.3)		837.2 (1353.0)	
Education			0.8895		0.7534		0.8814
Secondary school or less	26	982.5 (2522.6)		1456.0 (2396.1)		846.1 (838.7)	
Some or completed trade/technical school or college	36	1652.6 (7491.1)		2837.4 (9796.7)		949.9 (1853.6)	
Some university or higher	33	887.0 (3896.6)		1569.1 (5537.3)		837.2 (1192.6)	
Marital status			0.5785		0.8197		0.8308
Not married	88	1160.3 (3735.0)		1817.2 (7119.1)		833.8 (1489.2)	
Married	7	616.6 (5038.3)		1317.0 (4865.3)		1035.2 (1085.0)	
Living with someone else			0.7248		0.9715		0.7171
No	80	1129.3 (3764.0)		1731.2 (7124.3)		781.8 (1600.1)	
Yes	15	942.8 (4024.0)		2777.3 (4865.3)		1035.2 (1079.6)	
Dog ownership			0.5570		0.8752		0.9120
No	84	1160.3 (4036.8)		1794.8 (7134.7)		830.2 (1396.0)	
Yes	11	887.0 (2538.0)		1753.6 (5488.9)		837.2 (1280.9)	
Walkability^b^			0.0182		0.0096		0.0116
Car dependent (0–49)	19	3018.3 (16101.5)		7982.4 (23676.8)		2037.2 (2720.9)	
Somewhat walkable (50–69)	24	1194.6 (2898.6)		1888.4 (6268.4)		1037.9 (1355.4)	
Very walkable (70–89)	27	1614.0 (4074.9)		2243.0 (7064.9)		751.0 (1268.4)	
Walker’s paradise (90–100)	25	605.4 (1830.6)		1008.9 (2615.6)		542.4 (773.5)	
Length of time in neighborhood			0.4415		0.6514		0.7263
≤ 2 years	27	1593.0 (4628.6)		1912.1 (7060.9)		958.4 (1592.3)	
Between 2 and up to 6 years	28	839.8 (5695.7)		1535.3 (9962.9)		794.1 (1476.8)	
Between 6 and up to 9 years	17	942.8 (3631.5)		2243.0 (6116.3)		752.4 (1498.6)	
> 9 years	23	770.4 (3672.6)		1096.2 (7321.2)		701.9 (1310.5)	
Valid driver’s license			0.0624		0.0501		0.0309
No	23	617.6 (2543.3)		1053.7 (3075.3)		544.5 (1013.9)	
Yes	72	1218.1 (4266.3)		1904.0 (6986.1)		942.5 (1529.9)	
Access to a vehicle			0.0061		0.0027		0.0010
No	37	617.6 (2002.8)		1008.9 (2928.5)		504.7 (900.9)	
Yes	56	1613.7 (4678.6)		2501.9 (7009.7)		997.4 (1511.6)	
Social support/companionship to go outside			0.5381		0.3413		0.2613
No	44	982.5 (3446.7)		1456.8 (5476.1)		741.0 (1265.3)	
Yes	51	1183.8 (3789.5)		1896.0 (7054.8)		958.4 (1532.4)	
Physical support to go outside			0.0537		0.0405		0.0510
No	51	887.0 (2731.0)		1305.4 (3766.4)		695.0 (893.0)	
Yes	44	1942.6 (8296.2)		2937.3 (11791.8)		1047.8 (1757.7)	
Like to walk outside^c^			0.8864		0.9967		0.8929
Less than very much	28	813.5 (4273.7)		1222.3 (6828.0)		741.7 (1469.7)	
Very much	67	1136.8 (3683.4)		1896.0 (7124.4)		854.9 (1402.9)	
Confidence walking outside^c^			0.6580		0.7116		0.8444
Less than very much	20	684.3 (6950.3)		1168.8 (11618.3)		716.5 (1919.9)	
Very much	75	1183.8 (3482.5)		1896.0 (6947.3)		849.2 (1355.9)	
Falls in past 6 months			0.8301		0.8444		0.9164
No	75	1252.4 (3900.8)		1952.4 (7118.7)		854.9 (1389.1)	
Yes	20	1004.4 (3384.5)		1596.8 (6563.9)		824.3 (1407.8)	
Use of a mobility aid for walking			0.5248		0.5185		0.3691
No	78	1152.8 (3596.6)		1888.4 (7125.3)		852.1 (1421.1)	
Yes	17	1002.7 (6047.0)		1241.5 (9690.6)		752.4 (1556.3)	

*Abbreviations*: Interquartile range (IQR), Standard Deviation Ellipse using one standard deviation (SDE), Minimum Convex Polygon (MCP), Daily Path Area using a 200-meter buffer (DPA).

^a^p-value from Kruskal-Wallis non-parametric one-way Analysis of Variance (ANOVA) or Wilcoxon Rank Sum test across sociodemographic and resource categories.

^b^Measured by Street Smart Walk Score for home address.

^c^Less than very much (1–4 on a 5-point scale); Very much (5 on a 5-point scale).

Compactness values generated using SDE and MCP approaches were much higher than were DPA-generated values (Table 2), signifying more circular shapes by these approaches. Men had more compact SDE and evidence of more compact MCP, compared with women. Compactness values generated using SDE and MCP approaches were higher in areas with lower walkability (as measured by Walk Score), while compactness assessed using the DPA approach was higher in areas of higher walkability. Participants who had a valid driver’s license, and had access to a vehicle in the past 7 days had less compact values generated using the DPA approach. Participants who very much liked to walk outside had lower values for compactness if generated using SDE or MCP approaches.

**Table 2:** **GPS-based activity space compactness of Walk The Talk Study participants (n = 95) by sociodemographic group and resource characteristics**

Sociodemographic group	n	SDE compactness (0 to 1)		MCP compactness (0 to 1)		DPA compactness (0 to 1)	
		Mean (SD)	***p*** ^a^	Mean (SD)	***p*** ^a^	Mean (SD)	***p*** ^a^
All	95	0.77 (0.15)		0.75 (0.11)		0.34 (0.21)	
Sex			0.0506		0.0919		0.6597
Female	63	0.75 (0.15)		0.74 (0.11)		0.33 (0.21)	
Male	32	0.81 (0.12)		0.77 (0.09)		0.35 (0.21)	
Age (years)			0.9822		0.9412		0.2115
65-69	26	0.77 (0.14)		0.75 (0.10)		0.29 (0.21)	
70-74	29	0.77 (0.16)		0.74 (0.12)		0.31 (0.18)	
75-79	26	0.77 (0.15)		0.74 (0.11)		0.40 (0.23)	
80+	14	0.79 (0.13)		0.76 (0.09)		0.38 (0.21)	
Race			0.3052		0.3293		0.1429
Non-white	18	0.74 (0.12)		0.73 (0.11)		0.41 (0.27)	
White	77	0.78 (0.15)		0.75 (0.11)		0.33 (0.19)	
Education			0.1546		0.0172		0.4758
Secondary school or less	26	0.77 (0.16)		0.74 (0.11)		0.30 (0.11)	
Some or completed trade/technical school or college	36	0.81 (0.12)		0.79 (0.08)		0.37 (0.27)	
Some university or higher	33	0.74 (0.16)		0.71 (0.12)		0.34 (0.19)	
Marital status			0.8909		0.4912		0.9096
Not married	88	0.77 (0.15)		0.75 (0.11)		0.34 (0.21)	
Married	7	0.78 (0.15)		0.78 (0.10)		0.35 (0.25)	
Living with someone else			0.8379		0.5398		0.8590
No	80	0.77 (0.14)		0.75 (0.11)		0.34 (0.21)	
Yes	15	0.77 (0.16)		0.76 (0.10)		0.33 (0.21)	
Dog Ownership			0.9119		0.7896		0.6049
No	84	0.77 (0.14)		0.75 (0.11)		0.34 (0.20)	
Yes	11	0.77 (0.19)		0.76 (0.12)		0.37 (0.29)	
Walkability^b^			0.0721		0.0325		0.0244
Car dependent (0–49)	19	0.77 (0.13)		0.73 (0.11)		0.24 (0.14)	
Somewhat walkable (50–69)	24	0.83 (0.13)		0.79 (0.08)		0.31 (0.18)	
Very walkable (70–89)	27	0.77 (0.14)		0.77 (0.09)		0.36 (0.23)	
Walker’s paradise (90–100)	25	0.72 (0.16)		0.70 (0.13)		0.42 (0.23)	
Length of time in neighborhood			0.0659		0.4341		0.5547
≤ 2 years	27	0.77 (0.15)		0.75 (0.10)		0.29 (0.18)	
Between 2 and up to 6 years	28	0.76 (0.12)		0.74 (0.11)		0.36 (0.24)	
Between 6 and up to 9 years	17	0.71 (0.16)		0.72 (0.11)		0.34 (0.21)	
> 9 years	23	0.83 (0.14)		0.77 (0.11)		0.37 (0.21)	
Valid driver’s license			0.2126		0.9521		0.0175
No	23	0.74 (0.14)		0.75 (0.10)		0.43 (0.26)	
Yes	72	0.78 (0.15)		0.75 (0.11)		0.31 (0.19)	
Access to a vehicle			0.5099		0.7018		0.0007
No	37	0.76 (0.15)		0.75 (0.11)		0.43 (0.25)	
Yes	56	0.78 (0.14)		0.74 (0.11)		0.28 (0.16)	
Social support/companionship to go outside			0.5297		0.9556		0.5838
No	44	0.78 (0.15)		0.75 (0.11)		0.35 (0.21)	
Yes	51	0.76 (0.14)		0.75 (0.10)		0.33 (0.21)	
Physical support to go outside			0.3954		0.3574		0.1288
No	51	0.78 (0.14)		0.76 (0.10)		0.37 (0.22)	
Yes	44	0.76 (0.15)		0.74 (0.11)		0.31 (0.19)	
Like to walk outside^c^			0.0021		0.0037		0.9144
Less than very much	28	0.84 (0.11)		0.80 (0.08)		0.34 (0.22)	
Very much	67	0.74 (0.15)		0.73 (0.11)		0.34 (0.21)	
Confidence walking outside^c^			0.8907		0.7597		0.4028
Less than very much	20	0.78 (0.13)		0.74 (0.09)		0.38 (0.26)	
Very much	75	0.77 (0.15)		0.75 (0.11)		0.33 (0.19)	
Falls in past 6 months			0.8087		0.7402		0.7583
No	75	0.77 (0.15)		0.75 (0.11)		0.34 (0.21)	
Yes	20	0.77 (0.15)		0.74 (0.11)		0.33 (0.20)	
Use of a mobility aid for walking			0.3118		0.9095		0.1527
No	78	0.78 (0.14)		0.75 (0.11)		0.35 (0.22)	
Yes	17	0.74 (0.15)		0.75 (0.11)		0.27 (0.12)	

*Abbreviations*: Standard Deviation (SD), Standard Deviation Ellipse using one standard deviation (SDE), Minimum Convex Polygon (MCP), Daily Path Area using a 200-meter buffer (DPA).

^a^p-value from one-way Analysis of Variance (ANOVA) across sociodemographic and resource categories.

^b^Measured by Street Smart Walk Score for home address.

^c^Less than very much (1–4 on a 5-point scale); Very much (5 on a 5-point scale).

Log-linear models demonstrated that participants who lived in less walkable neighborhoods, who had access to a vehicle or who had physical support to go outside had significantly larger activity spaces (Table 3). Linear models demonstrated limited associations between sociodemographic or resource characteristics and compactness of activity space. Only three sociodemographic or resource characteristics were associated with compactness of activity spaces: living in walkable neighborhoods, liking to walk outside, and access to a vehicle. Using the SDE and MCP approaches, participants who lived in somewhat walkable neighborhoods had higher values for compactness and those who reported they very much like to walk outside had less compact activity spaces. Participants living in less walkable neighborhoods and with access to a vehicle had less compact DPA-generated activity spaces.

**Table 3:** **Associations between sociodemographic groups, resource characteristics and GPS-based activity space area and compactness of Walk The Talk Study Participants (n = 95)**

	Area			Compactness		
	SDE	MCP	DPA	SDE	MCP	DPA
	Percent difference (95% CL)	Percent difference (95% CL)	Percent difference (95% CL)	Mean difference (95% CL)	Mean difference (95% CL)	Mean difference (95% CL)
Male	−25.5 (−69.8, 83.6)	−19.0 (−66.6, 96.6)	−15.0 (−47.3, 37.2)	0.03 (−0.03, 0.09)	0.02 (−0.02, 0.07)	0.02 (−0.07, 0.11)
Age (years)^a^						
65-69	125.8 (−43.4, 801.1)	91.7 (−50.8, 647.7)	46.1 (−30.0, 204.5)	−0.07 (−0.17, 0.02)	−0.04 (−0.12, 0.03)	−0.08 (−0.22, 0.07)
70-74	71.1 (−54.5, 543.6)	63.4 (−55.6, 501.1)	30.8 (−35.3, 164.2)	−0.06 (−0.16, 0.03)	−0.05 (−0.12, 0.01)	−0.06 (−0.20, 0.08)
75-79	−43.3 (−85.0, 114.5)	−40.3 (−83.9, 120.7)	−17.1 (−59.1, 68.0)	−0.08 (−0.17, 0.02)	−0.05 (−0.12, 0.01)	0.03 (−0.11, 0.17)
Education^a^						
Some or completed trade/technical school or college	−15.2 (−69.9, 138.6)	−4.5 (−65.5, 164.1)	−1.9 (−43.4, 69.9)	0.05 (−0.02, 0.12)	**0.06 (0.00, 0.11)**	0.05 (−0.06, 0.15)
Some university or higher	−5.6 (−67.3, 172.5)	0.7 (−64.5, 185.7)	5.1 (−40.1, 84.6)	−0.01 (−0.08, 0.07)	−0.02 (−0.07, 0.04)	0.02 (−0.09, 0.13)
Walkability^a^						
Car Dependent (0–49)	**447.8 (54.8, 1838.7)**	**525.2 (80.4, 2066.1)**	**146.4 (26.0, 381.9)**	0.02 (−0.07, 0.11)	0.01 (−0.06, 0.07)	**−0.16 (−0.30, −0.03)**
Somewhat Walkable (50–69)	**213.7 (0.6, 877.8)**	**237.7 (10.4, 932.7)**	**102.3 (10.6, 269.9)**	**0.09 (0.01, 0.17)**	**0.07 (0.01, 0.13)**	−0.11 (−0.23, 0.01)
Very Walkable (70–89)	**225.5 (7.4, 886.6)**	**267.0 (23.3, 991.7)**	**95.8 (8.7, 252.7)**	0.03 (−0.05, 0.11)	0.05 (−0.01, 0.10)	−0.09 (−0.21, 0.02)
Length of time in Neighborhood^a^						
Between 2 and up to 6 years	------^b^	------^b^	------^b^	0.00 (−0.07, 0.08)	0.00 (−0.06, 0.05)	0.04 (−0.07, 0.15)
Between 6 and up to 9 years	------^b^	------^b^	------^b^	−0.06 (−0.15, 0.03)	−0.03 (−0.09, 0.03)	−0.03 (−0.16, 0.10)
> 9 years	------^b^	------^b^	------^b^	0.06 (−0.02, 0.14)	0.02 (−0.04, 0.07)	0.03 (−0.08, 0.15)
Have a valid driver’s license	46.4 (−62.0, 463.3)	50.5 (−60.0, 466.4)	17.7 (−42.4, 140.8)	0.03 (−0.07, 0.12)	−0.01 (−0.08, 0.06)	−0.01 (−0.15, 0.13)
Have access to a vehicle	**285.1 (25.0, 1085.9)**	**304.4 (33.8, 1122.4)**	**139.8 (32.0, 335.6)**	−0.02 (−0.09, 0.06)	−0.02 (−0.07, 0.04)	**−0.16 (−0.27, −0.04)**
Have physical support to go outside	**184.2 (18.5, 581.2)**	**184.9 (20.6, 573.2)**	**75.0 (10.0, 178.4)**	−0.03 (−0.09, 0.03)	−0.02 (−0.06, 0.02)	−0.08 (−0.17, 0.01)
Like to walk outside very much	------^b^	------^b^	------^b^	**−0.09 (−0.15, −0.03)**	**−0.06 (−0.11, −0.02)**	−0.03 (−0.12, 0.06)

*Abbreviations*: Confidence Limits (CL), Standard Deviation Ellipse using one standard deviation (SDE), Minimum Convex Polygon (MCP), Daily Path Area using a 200-meter buffer (DPA). Bold values indicate estimates with p < 0.05.

^a^Reference categories: 80+ years old; secondary school or less; Walker’s Paradise (Walk Score 90–100); living in neighborhood less than 2 years.

^b^Not tested in models of area due to lack of significance in bivariate analysis.

## Discussion

This research is one of the first studies to utilize GPS tracking to create activity spaces as a means to assess older adult mobility. We extend the literature by demonstrating that GPS tracking can be used to create three different types of activity spaces as a valuable tools to better understand the geographic mobility patterns of older adults. Not surprisingly, older adults deemed most mobile based on their age, the walkability of their neighborhoods, and whether or not they drove, had the largest activity spaces. The trends in activity space areas were not different, regardless of what approach was used to generate them. However, shape of activity spaces (measured as compactness) varied by approach.

Walking and cycling trips are often extend beyond traditional buffer sizes (1 mile, ½ mile) used to represent neighborhoods where older adults live [56]. We demonstrated that GPS may enable a more precise way to operationalize neighborhoods than residential buffers or administrative units [51, 57–62] as it better captures the locations individuals actually visit rather than a presumed neighborhood boundary. GPS has recently become a more popular option for measuring neighborhood exposure and context in health studies [33, 50, 63–67]. As the analytic approaches to GPS data develop, this technology may become a powerful tool to accurately and precisely describe people’s interactions with geographic space, including older adult mobility. It is possible that different types of activity spaces may be more appropriate depending on the application or the research question being addressed. Since the MCP-generated activity space is bounded by the outermost GPS points, it captures the envelope of the extreme extent of travel and thus may include large geographic areas that are not visited by, or important to, an individual [68]. In contrast, SDE-generated spaces may be more useful if one wishes to assess the direction and general shape of a person’s travel area, without introducing potential error introduced by using geographically distant points. In addition, SDE approaches may indicate the frequency that an individual visits a geographic area as more points are generated in that location - this ‘pulls’ the SDE-generated activity space toward that more often visited geographic area. However, since SDE-generated activity spaces are by definition ellipse shaped, they may also capture a substantial area that an individual may not have visited. Finally, we recommend using DPA to generate an activity space if a research question is about the destinations participants pass in daily travel, as it relies solely on buffering the routes actually traveled.

The consistency of activity space area across the three different approaches aligns with a previous study that showed associations across MCP, SDE, and line-based buffers [52]. However, no previous studies assessed the shape (compactness) of GPS-derived activity spaces. In our study, patterns of compactness differed depending on the approach used to generate activity space. This signals the need to choose an approach to generating an activity space that is relevant to the research question being asked. Of interest, compactness generated using the SDE and MCP approach was higher when walkability was lower, whereas for DPA-generated compactness was higher in neighborhoods with higher Walk Score. This finding may illustrate that participants in neighborhoods with lower walkability are clustering their trips to a nearby retail area. Thus, in neighborhoods with lower walkability the area of all three activity space types becomes larger, and the compactness of the SDE and MCP is higher. However, in this same scenario, DPA-generated activity spaces become more elongated, potentially with multiple trips heading in the same direction, which results in a less compact DPA-generated activity space. Our findings using DPA are consistent with work indicating that small and compact activity spaces increase the likelihood of walking and cycling [55].

A number of factors surfaced as important to older adults’ mobility. First, it is intuitive and supported by previous studies that an older adult’s ability to drive will influence the size of their activity space [23–26], and that the activity space of older adult will be smaller compared with a younger person, on average [22]. Sex did not surface as a differentiating factor in our study, in contrast with previous reports that women had smaller activity spaces than did men [22, 23]. However, as only one-third of our sample were men it is possible we lacked the statistical power to test this association. Second, when an older person is no longer able to drive, physical support to maintain mobility within the neighborhood becomes increasingly important. Given its apparent role in our study around encouraging older adults to travel within their neighborhoods, physical support may represent an effective intervention in future studies. Third, our results support previous findings – that neighborhood attributes (i.e. higher street connectivity, proximity to destinations, and traffic conditions, and parks) are associated with increased mobility among older adults [65, 69–71]. Although seemingly counterintuitive at first glance, our finding that those living in higher walkability areas had smaller activity spaces could reflect closer proximity and access to amenities, and the use of different modes of transport. That is, highly walkable neighborhoods are more likely to have destinations that older people deem important, within easier walking access. As walking trips are often shorter than driving trips, this would reduce distance traveled to these destinations. However, this study cannot disentangle the competing and related concepts of walkability and car usage. On one hand, car usage was associated with larger activity spaces, potentially indicating greater mobility, yet walkability was associated with smaller spaces, potentially indicating that the local neighborhood environment is sufficient to fulfill daily activities and amenities. Future work creating activity spaces by mode or considering the distance traveled or frequency of trips within an activity space may help tease apart these complex and complementary elements of older adult mobility. All of these results are novel and in our view, are worth pursuing in future trials that evaluate different groups of older adults (we recruited older adults with low incomes) who reside across diverse built environment settings.

The life-space literature indicates that greater mobility is related to a wide range of favorable health outcomes [18–21]. This study indicates that larger activity spaces are associated with a number of different resources such as younger age, access to a vehicle, or physical support for going outside. One possibility is that the geographic mobility of individuals, as measured either in life-space or activity space, is in fact a proxy for personal resources. Additional work, examining changes in activity spaces as older adults transition through life changes (e.g. retirement, loss of a spouse, move to a more walkable neighborhood, driving cessation) may help to tease apart the complex connections between older adult resources, geographic mobility, and health outcomes.

### Strengths and limitations

Our study has a number of strengths, including the characterization of a sample of older adults with low income, the examination of multiple methods to create activity space, and the provision of sufficient code to utilize these methods in other studies. We acknowledge that our study also has several limitations. It was not possible to draw strong conclusions given the relatively small and select sample, the cross-sectional design, and potential measurement error associated with self-reported characteristics and trip identification. Although older adults may experience some discomfort while wearing GPS devices [72], they were also highly compliant in wearing them. There was no clear association between any characteristics of older adults and their level of compliance with wearing GPS [73]. This study did not examine specific destinations or resources within activity spaces, although work building on this can illuminate factors that contribute to the capacity of neighborhoods to provide opportunities to older adults. Finally, bias associated with selective daily mobility may be a barrier to causal inference when using GPS to assess neighborhood exposure [74].

## Conclusion

There are many different ways to represent geographic activity spaces where individuals travel to and spend their time. However, outcomes and interpretations may vary based on the approach used to generate an activity space. It is important to use an approach tailored to the needs of a specific research question and outcomes. Some factors we identified as important to geographic mobility of older adults may be used to inform interventions and to design policies that support older adults living and engaging independently with their community. Specifically, this work highlights the role of neighborhood walkability, driving patterns, and physical support to go outside as important factors in determining the size of older adult activity spaces. Identifying an approach that best captures the activity space of older adults may be useful for future work aimed at isolating features of the neighborhood environment that support older people ‘aging in place’ or informing interventions and policies that support older adults living independently in the community.

## Methods

### Sample

**Figure 2:**
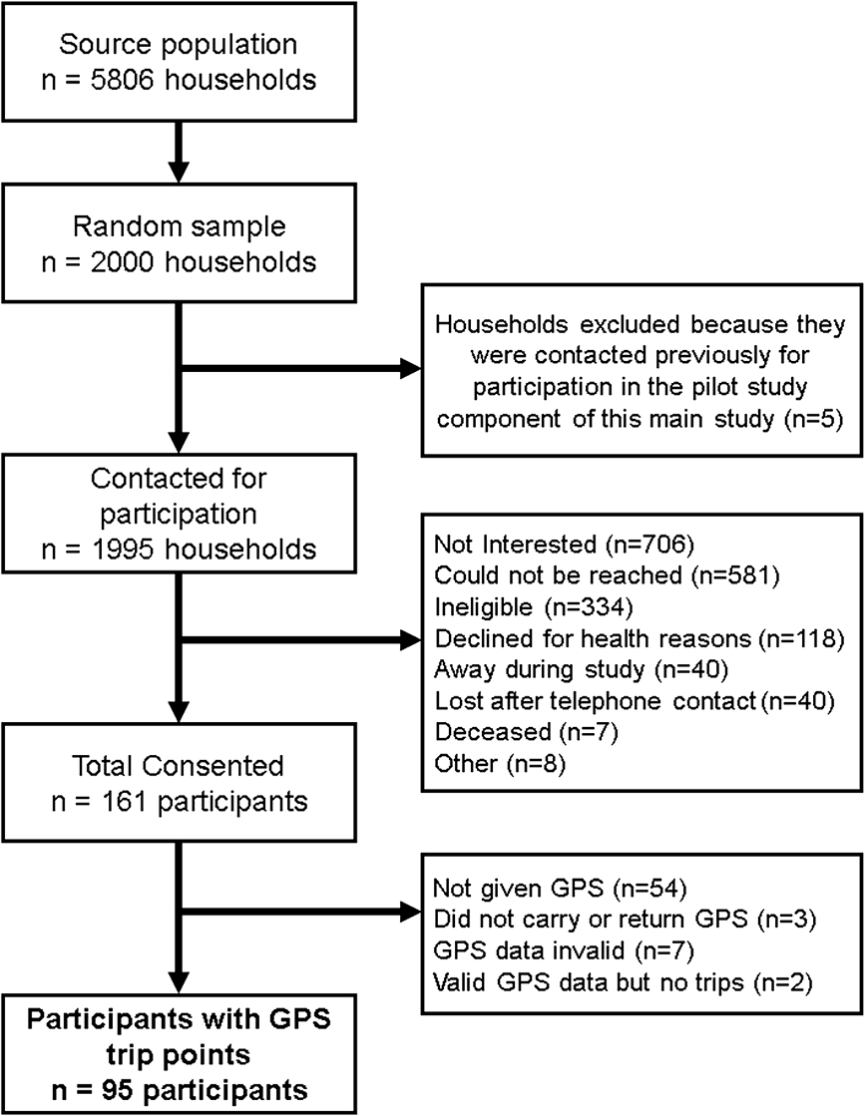
**Walk The Talk (WTT) Participant recruitment and flow for GPS data.** Source population comprised of households in our study area (Burnaby, New Westminster, North Vancouver, Richmond, Surrey, Vancouver, West Vancouver, White Rock) that receive a Shelter Aid for Elderly Renters rental subsidy from BC Housing, have a head of household aged greater than or equal to 65 years, and a telephone number on file with BC Housing. Participants were considered lost after telephone contact if they could not be reached again after expression of interest in study participation. GPS data was considered invalid if the unit was turned to the off position by the participant.

Participants were recruited to take part in Walk the Talk (WTT), a cross-sectional study (n = 161) that evaluates the association between the built environment and the mobility and health of low-income older adults. Participants reside in eight cities in Metropolitan Vancouver (Burnaby, New Westminster, North Vancouver, Richmond, Surrey, Vancouver, West Vancouver, White Rock). Methods for WTT are described elsewhere [75], but briefly: WTT base population consists of 5806 households that receive a Shelter Aid for Elderly Renters (SAFER) rental subsidy from BC Housing, had a head of household aged ≥ 65 years, and a telephone number on file with BC Housing (Figure 2). Households were sampled using a random stratified design, selecting 200 households from within each decile of walkability, measured using Street Smart Walk Score (http://www.walkscore.com) (n_total_ = 2000), to ensure that participants were recruited across a range of built environments. Recruitment was done via telephone between January and February 2012. Individuals were excluded if they were diagnosed with dementia, left their home less than once in a typical week, were unable to understand or speak English, were unable to walk more than ten meters with or without a mobility aid (e.g. cane, walker), or were unable to participate in a mobility assessment involving a four meter walk. Measurement was conducted between March and May 2012. At the end of the measurement sessions, participants were instructed regarding wear of accelerometers and completion of travel diaries. A sub-group (n = 107) of participants received GPS and were instructed as to their use. The study was approved by the University of British Columbia’s Clinical Research Ethics Board (certificate: H10-02913).

### Travel data

Home locations were geocoded based on participant reported home address. Participants’ travel patterns and physical activity was assessed using travel diaries, QStarz Datalogger BT-Q1000XT GPS sensors (Semsons, Arcadia, CA, USA; recording at 1 s) and ActiGraph GT3X-Plus tri-axial accelerometers (ActiGraph LLC, Fort Walton Beach, FL, USA), respectively, over the 7 days immediately following measurement sessions. For travel diaries, participants were instructed to record for each trip: start and end locations and times, reason for travel, mode of travel, and others who accompanied them. For the GPS sensors, the vibration sensor was activated to preserve memory and battery life; participants were not asked to charge devices so the data collection period was a function of battery life. GPS data were downloaded using the QStarz Data Viewer software. Of participants who were given GPS, 97.2% wore them and of these, we acquired valid data from 93.3% (n = 97). For accelerometry, data were downloaded using the ActiLife software.

There is little consensus regarding best-practices for processing GPS data [76]. Previously, customized automated algorithms were used to identify destinations and trips from GPS data [77–80]. However, to capitalize on data acquired from travel diaries and to address broader questions related to multi-modal trips, we coded GPS data manually for this study, as has been done by our team [81] and others [82]. In brief, the 1-sec GPS data were first time-aligned with accelerometer data and then processed using ArcGIS tracking analyst in concert with travel diaries to define the start and end points of trips based on trip speed, distance, duration, and accelerometry-defined activity level. Tracks had to be of ≥30 s in duration and ≥100 m distance to be considered a trip. Trip start was identified as the first GPS point outside of home or leaving the previous trips’ destination location where speed ≥1 km/h and distance >0 m of movement. Changes from these criteria indicated trip stop time, allowing for pauses of <5 min (e.g. at a stop light, bus stop). Two participants who did not log at least one out-of-home trip were excluded. Thus, the final sample size was n = 95 men and women who provided 333 days of recorded GPS data. We removed trips outside the metropolitan Vancouver area so as to represent participant movement within the region.

### GPS activity spaces

**Figure 3:**
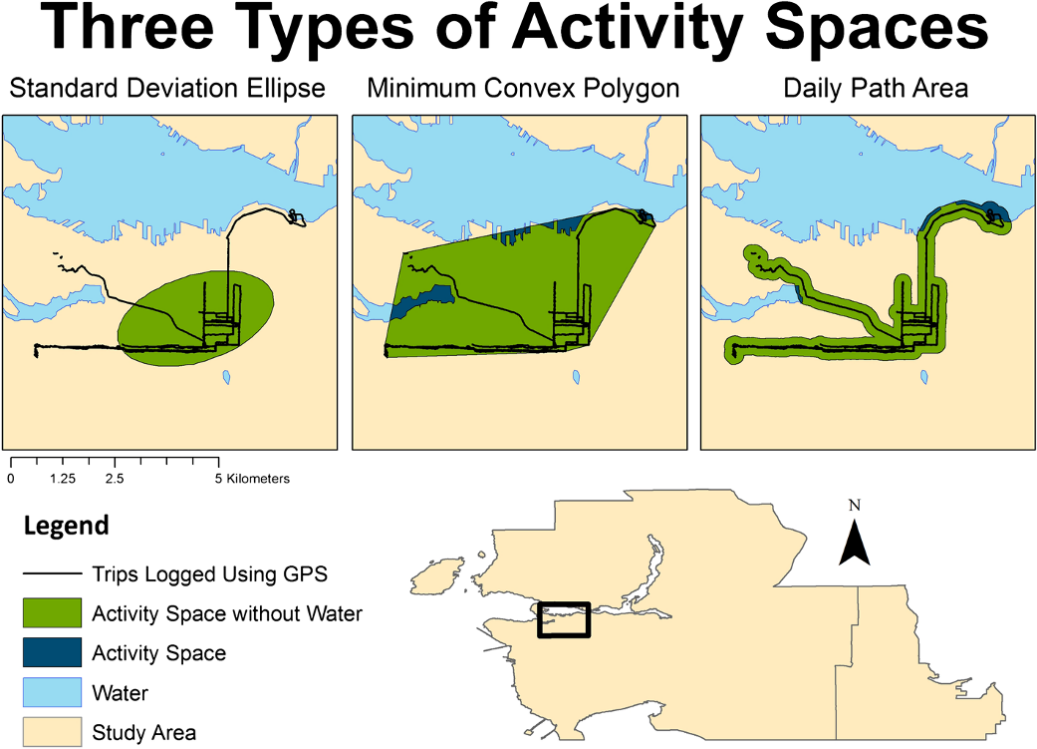
**Example of three types of activity spaces.**

There are a number of different ways, derived from geography and ecology, to analyze geographic behaviors using point data [67]. We analyzed trip-related GPS point data (n = 1,392,347), aggregated by individual, using Python 2.7.2 (Python Software Foundation, http://www.python.org) and ArcPy for ArcGIS 10.1 (ESRI, Redlands, CA, USA). We represented activity space using three different approaches for each participant. They were; 1. Standard Deviation Ellipse (SDE), 2. Minimum Convex Polygon (MCP), and 3. Daily Path Area (DPA) (Figure 3). SDE, a commonly used measures of activity space, measures the directional distribution of a series of points [49, 50, 52, 62, 83–85]. Similar to others [49, 50], we used a one-SDE that contains 68% of all GPS points. MCP, sometimes referred to as “home ranges”, represents the smallest polygon that contains all GPS points [62, 86], with the outermost points serving as vertices [68]. DPA were adapted from previous literature [50, 52, 87]. We created them by buffering all of an individual’s trips by 200-meters. We conducted sensitivity analyses on activity spaces with and without water. Areas were highly correlated (Spearman’s ρ >0.99, p < 0.0001) and results were consistent across measures with and without water (not presented).

For the three activity space polygons we calculated two dimensions of activity space: 1. area (hectares) and 2. compactness. Area and perimeter were generated using “Calculate Geometry” in ArcGIS. Compactness is a measure of how circular a polygon is; a value near 1 indicates the activity space is similar to a circle while a value near 0 indicates an elongated space, more closely resembling a line [53, 55]. Compactness is calculated as the ratio of the perimeter of a circle with the same area to perimeter of the observed activity space. Compactness values may be related to the activity space approach (e.g., SDE would be expected to be more compact that DPA), however, within a given activity space type the comparison of compactness across individual sociodemographic and resource characteristics can highlight determinants of the shape, or local orientation, of travel.

We provide python code to create area and compactness across the three activity space measures, both with and without water, in the Additional file 1 that supports this paper.

### Sociodemographic and resource characteristics

Participants self-reported sociodemographic and resource characteristics during measurement sessions. Self-reported age (65–69 years; 70–74 years; 75–79 years; 80+ years), race (White; non-White), education level (completed secondary school or less; some trade/technical school or college through completed trade/technical school or college diploma; some university or higher), marital status (single; married; widowed, separated or divorced), cohabitation with someone, dog ownership, current valid driver’s license, and vehicle at their disposal were assessed via questionnaire. We assessed neighborhood walkability using Street Smart Walk Score, a single measure that accounts for distance to popular amenities and street design (http://www.walkscore.com). We categorized walkability based on cut-off categories as recommended by designers of Street Smart Walk Score (car dependent 0–49, somewhat walkable 50–69, very walkable 70–89, walker’s paradise 90–100). In-depth description of Walk Score can be found elsewhere [88, 89]. Participants also reported how long they lived in their current neighborhood (classified into quartiles: less than 2 years; between 2 and up to 6 years; between 6 and up to 9 years; more than 9 years), whether people in their lives offered support related to going outside in their neighborhood (no or don’t know; yes, people that offer physical support (drive places); yes, people that offer social support/companionship; yes, people that offer both physical and social support), how much they liked to walk outside (not at all, not much or neutral; somewhat; very much), how confident they were walking in their neighborhood (not at all, not much or neutral; somewhat; very much), and whether they had any falls in the past 6 months or used a mobility aid for walking.

### Statistical methods

We examined the correlation between each of the three activity spaces using Spearman’s and Pearson’s correlation coefficients (ρ) for area and compactness, respectively. We describe area using medians and IQR due to non-normal distribution. We describe compactness as mean and SD. We used one-way Analysis of Variance (ANOVA), Kruskal-Wallis non-parametric one-way ANOVA or Wilcoxon Rank Sum test to test for differences in area and compactness of each activity space across sociodemographic and resource categories, as appropriate. We used linear Ordinary Least Squares (OLS) regression models, to assess the associations between sociodemographic and resource characteristics of participants and log-transformed area or non-transformed compactness of activity spaces after adjustment for other potential variables. Variables were included in the simultaneous model based on a-priori hypothesis (sex, age, education) or if they were associated (p < 0.1) to dependent variables in bivariate analyses. To enhance interpretability, results from regression models for area have been retransformed and presented as percentage differences. We conducted all statistical analyses using SAS software, Version 9.3 (SAS Institute Inc., Cary, NC, USA).

## Electronic supplementary material

Additional file 1: **We have provided a generic python script to enable others to easily create activity spaces using their own GPS data: Activity_Space_Processing_TEMPLATE_supplement.py.** (ZIP 3 KB)

## Abbreviations

ANOVA: Analysis of variance
DPA: Daily path area
GPS: Global positioning system
IQR: Interquartile range
MCP: Minimum convex polygon
SAFER: Shelter aid for elderly renters
SenTra: Senior tracking
SD: Standard deviation
SDE: Standard deviation ellipse
WTT: Walk the talk.
